# Supplementary material for: Unveiling the Bioactive Potential of the Invasive Jellyfish Phyllorhiza punctata Through Integrative Transcriptomic and Proteomic Analyses
Source: Biomolecules. 2025 Aug 4;15(8):1121. doi: 10.3390/biom15081121 (PMC12383608; doi:10.3390/biom15081121)
Supplement: Supplementary file 1 [file biomolecules-15-01121-s001.zip › Suplementary Material/FileS3/PEPFOLD4/XP_065063801_15156/clusters.html]

 PEP-FOLD clusters


Download archive of all models  here   

|  |  |  |
| --- | --- | --- |
| # | file | sOPEP |
| # cluster 1 - Size: 21 |  |  |
|  | XP\_065063801\_15156-model1 | -8.26926 |
|  | XP\_065063801\_15156-model1.1 | -8.13669 |
|  | XP\_065063801\_15156-model1.2 | -8.11921 |
|  | XP\_065063801\_15156-model1.3 | -8.10648 |
|  | XP\_065063801\_15156-model1.4 | -8.00333 |
|  | XP\_065063801\_15156-model1.5 | -7.87479 |
|  | XP\_065063801\_15156-model1.6 | -7.85682 |
|  | XP\_065063801\_15156-model1.7 | -7.78591 |
|  | XP\_065063801\_15156-model1.8 | -7.76216 |
|  | XP\_065063801\_15156-model1.9 | -7.72057 |
|  | XP\_065063801\_15156-model1.10 | -7.71993 |
|  | XP\_065063801\_15156-model1.11 | -7.7029 |
|  | XP\_065063801\_15156-model1.12 | -7.68666 |
|  | XP\_065063801\_15156-model1.13 | -7.68499 |
|  | XP\_065063801\_15156-model1.14 | -7.66585 |
|  | XP\_065063801\_15156-model1.15 | -7.60417 |
|  | XP\_065063801\_15156-model1.16 | -7.60013 |
|  | XP\_065063801\_15156-model1.17 | -7.59757 |
|  | XP\_065063801\_15156-model1.18 | -7.54785 |
|  | XP\_065063801\_15156-model1.19 | -7.52979 |
|  | XP\_065063801\_15156-model1.20 | -6.71116 |
| # cluster 2 - Size: 4 |  |  |
|  | XP\_065063801\_15156-model2 | -7.93395 |
|  | XP\_065063801\_15156-model2.1 | -7.4575 |
|  | XP\_065063801\_15156-model2.2 | -7.44146 |
|  | XP\_065063801\_15156-model2.3 | -6.87308 |
| # cluster 3 - Size: 4 |  |  |
|  | XP\_065063801\_15156-model3 | -7.9094 |
|  | XP\_065063801\_15156-model3.1 | -7.59258 |
|  | XP\_065063801\_15156-model3.2 | -6.98356 |
|  | XP\_065063801\_15156-model3.3 | -4.45901 |
| # cluster 4 - Size: 2 |  |  |
|  | XP\_065063801\_15156-model4 | -4.6228 |
|  | XP\_065063801\_15156-model4.1 | -4.41404 |
| # cluster 5 - Size: 3 |  |  |
|  | XP\_065063801\_15156-model5 | -4.49988 |
|  | XP\_065063801\_15156-model5.1 | -4.3909 |
|  | XP\_065063801\_15156-model5.2 | -4.35218 |
| # cluster 6 - Size: 7 |  |  |
|  | XP\_065063801\_15156-model6 | -4.48321 |
|  | XP\_065063801\_15156-model6.1 | -4.47478 |
|  | XP\_065063801\_15156-model6.2 | -4.45897 |
|  | XP\_065063801\_15156-model6.3 | -4.38695 |
|  | XP\_065063801\_15156-model6.4 | -4.32817 |
|  | XP\_065063801\_15156-model6.5 | -4.32763 |
|  | XP\_065063801\_15156-model6.6 | -4.23854 |
| # cluster 7 - Size: 1 |  |  |
|  | XP\_065063801\_15156-model7 | -4.47711 |
| # cluster 8 - Size: 10 |  |  |
|  | XP\_065063801\_15156-model8 | -4.47595 |
|  | XP\_065063801\_15156-model8.1 | -4.47128 |
|  | XP\_065063801\_15156-model8.2 | -4.44173 |
|  | XP\_065063801\_15156-model8.3 | -4.38065 |
|  | XP\_065063801\_15156-model8.4 | -4.34581 |
|  | XP\_065063801\_15156-model8.5 | -4.30918 |
|  | XP\_065063801\_15156-model8.6 | -4.29479 |
|  | XP\_065063801\_15156-model8.7 | -4.28487 |
|  | XP\_065063801\_15156-model8.8 | -4.27923 |
|  | XP\_065063801\_15156-model8.9 | -4.22255 |
| # cluster 9 - Size: 5 |  |  |
|  | XP\_065063801\_15156-model9 | -4.47509 |
|  | XP\_065063801\_15156-model9.1 | -4.43757 |
|  | XP\_065063801\_15156-model9.2 | -4.43085 |
|  | XP\_065063801\_15156-model9.3 | -4.41694 |
|  | XP\_065063801\_15156-model9.4 | -4.2886 |
| # cluster 10 - Size: 8 |  |  |
|  | XP\_065063801\_15156-model10 | -4.47435 |
|  | XP\_065063801\_15156-model10.1 | -4.35406 |
|  | XP\_065063801\_15156-model10.2 | -4.34084 |
|  | XP\_065063801\_15156-model10.3 | -4.32635 |
|  | XP\_065063801\_15156-model10.4 | -4.31612 |
|  | XP\_065063801\_15156-model10.5 | -4.29587 |
|  | XP\_065063801\_15156-model10.6 | -4.28161 |
|  | XP\_065063801\_15156-model10.7 | -4.23665 |
| # cluster 11 - Size: 4 |  |  |
|  | XP\_065063801\_15156-model11 | -4.47312 |
|  | XP\_065063801\_15156-model11.1 | -4.44194 |
|  | XP\_065063801\_15156-model11.2 | -4.4273 |
|  | XP\_065063801\_15156-model11.3 | -4.2276 |
| # cluster 12 - Size: 2 |  |  |
|  | XP\_065063801\_15156-model12 | -4.45191 |
|  | XP\_065063801\_15156-model12.1 | -4.40623 |
| # cluster 13 - Size: 1 |  |  |
|  | XP\_065063801\_15156-model13 | -4.45154 |
| # cluster 14 - Size: 7 |  |  |
|  | XP\_065063801\_15156-model14 | -4.44078 |
|  | XP\_065063801\_15156-model14.1 | -4.40122 |
|  | XP\_065063801\_15156-model14.2 | -4.39168 |
|  | XP\_065063801\_15156-model14.3 | -4.32806 |
|  | XP\_065063801\_15156-model14.4 | -4.3273 |
|  | XP\_065063801\_15156-model14.5 | -4.32695 |
|  | XP\_065063801\_15156-model14.6 | -4.30571 |
| # cluster 15 - Size: 1 |  |  |
|  | XP\_065063801\_15156-model15 | -4.4336 |
| # cluster 16 - Size: 1 |  |  |
|  | XP\_065063801\_15156-model16 | -4.39402 |
| # cluster 17 - Size: 6 |  |  |
|  | XP\_065063801\_15156-model17 | -4.37186 |
|  | XP\_065063801\_15156-model17.1 | -4.32345 |
|  | XP\_065063801\_15156-model17.2 | -4.29701 |
|  | XP\_065063801\_15156-model17.3 | -4.29178 |
|  | XP\_065063801\_15156-model17.4 | -4.29178 |
|  | XP\_065063801\_15156-model17.5 | -4.23413 |
| # cluster 18 - Size: 1 |  |  |
|  | XP\_065063801\_15156-model18 | -4.35106 |
| # cluster 19 - Size: 2 |  |  |
|  | XP\_065063801\_15156-model19 | -4.3366 |
|  | XP\_065063801\_15156-model19.1 | -4.31669 |
| # cluster 20 - Size: 2 |  |  |
|  | XP\_065063801\_15156-model20 | -4.29676 |
|  | XP\_065063801\_15156-model20.1 | -4.29407 |
| # cluster 21 - Size: 1 |  |  |
|  | XP\_065063801\_15156-model21 | -4.29122 |
| # cluster 22 - Size: 3 |  |  |
|  | XP\_065063801\_15156-model22 | -4.29106 |
|  | XP\_065063801\_15156-model22.1 | -4.28672 |
|  | XP\_065063801\_15156-model22.2 | -4.257 |
| # cluster 23 - Size: 1 |  |  |
|  | XP\_065063801\_15156-model23 | -4.29096 |
| # cluster 24 - Size: 2 |  |  |
|  | XP\_065063801\_15156-model24 | -4.27692 |
|  | XP\_065063801\_15156-model24.1 | -4.20908 |
| # cluster 25 - Size: 1 |  |  |
|  | XP\_065063801\_15156-model25 | -4.20731 |
|  |  |  |
